# Growth-Restricted Fetuses and Offspring Reveal Adverse Sex-Specific Metabolic Responses in Preeclamptic Mice Expressing Human sFLT1

**DOI:** 10.3390/ijms24086885

**Published:** 2023-04-07

**Authors:** Rebekka Vogtmann, Mian Bao, Monia Vanessa Dewan, Alina Riedel, Rainer Kimmig, Ursula Felderhoff-Müser, Ivo Bendix, Torsten Plösch, Alexandra Gellhaus

**Affiliations:** 1 Department of Gynecology and Obstetrics, University Hospital Essen, 45147 Essen, Germanyalina.riedel@uk-essen.de (A.R.); rainer.kimmig@uk-essen.de (R.K.); 2 Department of Obstetrics and Gynecology, University of Groningen, University Medical Center Groningen, 9713 GZ Groningen, The Netherlands; m.bao@umcg.nl (M.B.); torsten.ploesch@uni-oldenburg.de (T.P.); 3 Department of Pediatrics I, Neonatology & Experimental Perinatal Neurosciences, University Hospital Essen, Centre for Translational Neuro- and Behavioural Sciences, C-TNBS, Faculty of Medicine, University Duisburg-Essen, 45147 Essen, Germany; monia.dewan@uk-essen.de (M.V.D.); ursula.felderhoff@uk-essen.de (U.F.-M.); ivo.bendix@uk-essen.de (I.B.); 4 Perinatal Neurobiology Research Group, School of Medicine and Health Sciences, Carl von Ossietzky University of Oldenburg, 26111 Oldenburg, Germany

**Keywords:** preeclampsia, fetal growth restriction, sFLT1, liver, placenta, fetal programming, sex differences, obesity

## Abstract

Fetal adaptations to harmful intrauterine environments due to pregnancy disorders such as preeclampsia (PE) can negatively program the offspring’s metabolism, resulting in long-term metabolic changes. PE is characterized by increased circulating levels of sFLT1, placental dysfunction and fetal growth restriction (FGR). Here we examine the consequences of systemic human sFLT1 overexpression in transgenic PE/FGR mice on the offspring’s metabolic phenotype. Histological and molecular analyses of fetal and offspring livers as well as examinations of offspring serum hormones were performed. At 18.5 dpc, sFLT1 overexpression resulted in growth-restricted fetuses with a reduced liver weight, combined with reduced hepatic glycogen storage and histological signs of hemorrhages and hepatocyte apoptosis. This was further associated with altered gene expression of the molecules involved in fatty acid and glucose/glycogen metabolism. In most analyzed features males were more affected than females. The postnatal follow-up revealed an increased weight gain of male PE offspring, and increased serum levels of Insulin and Leptin. This was associated with changes in hepatic gene expression regulating fatty acid and glucose metabolism in male PE offspring. To conclude, our results indicate that sFLT1-related PE/FGR in mice leads to altered fetal liver development, which might result in an adverse metabolic pre-programming of the offspring, specifically targeting males. This could be linked to the known sex differences seen in PE pregnancies in human.

## 1. Introduction

The well-accepted fetal programming hypothesis [1] known as “Developmental Origins of Health and Disease” (DOHaD) presumes that an impaired intrauterine environment due to insufficient or excess nutrition as well as the exposure to adverse stimuli can influence fetal physiology, thereby affecting long-term offspring health [2]. Many studies have shown that such circumstances and placental diseases such as preeclampsia (PE) negatively affect fetal and offspring birth weight and lead to cardiovascular, neurodevelopmental as well as metabolic disorders later in life [3,4,5]. Metabolic syndrome, obesity and diabetes in mothers are risk factors of PE, which conversely can also cause these metabolic diseases in the offspring [6,7]. PE is a placental disorder, affecting 3–7% of pregnant women worldwide, leading to high rates of mortality and morbidity, and is often associated with fetal growth restriction (FGR) and preterm birth [8,9]. Preeclamptic patients who suffered from hypertension combined with further secondary organ failures exhibit elevated serum levels of circulating anti-angiogenic soluble fms-like tyrosine kinase-1 (sFLT1). PE often originates in the first trimester of pregnancy due to a combination of various risk factors such as genetic predispositions, oxidative stress or immunological factors, e.g., autoantibodies or dysfunctional uterine natural killer cells. The multifactorial disease PE ultimately leads to a dysfunctional placenta, which secretes anti-angiogenic factors, further resulting in the clinical manifestations of PE later on in pregnancy [10]. One of these factors thought to be produced by the dysfunctional placenta is sFLT1, which accumulates in maternal serum with ongoing pregnancy [11] and is used as the predictive biomarker in clinical practice [12]. sFLT1 serves as an antagonist to placental growth factor (PlGF) and vascular endothelial growth factor (VEGF), promoting maternal hypovascularization and endothelial dysfunction [13]. Recent studies by us and others suggest that sFLT1 overexpression leads to placental insufficiency and FGR, and further impairs the placental metabolic processes and thus might have also a negative impact on the fetal and offspring’s postnatal health [7,14,15,16,17,18,19].

Previously, we have generated a transgenic mouse model for preeclampsia by the systemic overexpression of human sFLT1 (hsFLT1), one of the driving factors in human PE and used as a predictive clinical biomarker [15,20]. In this model, dams exhibited preeclampsia-like symptoms as observed in the clinic, e.g., new-onset hypertension, combined with glomerular endotheliosis and proteinuria. Furthermore, upon hsFLT1 overexpression and elevated secretion of sFLT1 levels in the maternal circulation, a dysfunctional placenta with an impaired utero-placental vascularization was shown, resulting in growth-restricted fetuses [20]. Thus, this PE mouse model phenocopies all clinical symptoms of PE, only by increasing the anti-angiogenic molecule hsFLT1. Additionally, a reduced expression of placental nutrient transporters was detected [15]. However, the specific function of hsFLT1 on fetal physiology and organ development remains an open question. Quite recently we observed a reduced cortical expansion in fetal brains upon hsFLT1 overexpression [21]. We hypothesized that increased anti-angiogenesis by hsFLT1 overexpression not only negatively affects fetal brain development, but might also impair the development of other fetal organs. Together with the previously shown metabolic changes in hsFLT1-affected placentas [15] and disrupted blood and possibly nutrient supply to hsFLT1-affected fetuses [20], we also expect metabolic maladaptations in the offspring of PE/FGR mice. It is well known that previous growth-restricted children and the offspring of PE pregnancies have a higher risk of developing metabolic diseases or obesity [22]. The liver is a highly metabolic active organ, with the main functions such as bile production, the excretion of cholesterol and hormones, the metabolism of fatty acids, proteins and carbohydrates including the storage and homeostasis of glycogen, vitamins and minerals and the synthesis of plasma proteins and clotting factors [23]. During fetal development, the liver also represents the only contact between the developing placental vessels and the fetal heart. Furthermore, the liver functions as a hematopoietic tissue during fetal development, which produced blood stem cells prior to bone marrow development [24]. Thus, defects in liver function during fetal development can have severe consequences for the fetal well-being during pregnancy and can have further metabolic consequences for the offspring. Consequently, we aim to characterize liver morphology at the end of the pregnancy in the fetus as well as in the offspring.

To gain further insight into the impact of hsFLT1 overexpression on the offspring’s metabolic phenotype, we investigated the effects of systemic hsFLT1 overexpression on the mice offspring’s weight gain and obesity-related phenotype. Specifically, we characterized the influence of hsFLT1 on fetal (18.5 dpc) and adult (P90) liver development and on important metabolic pathways in the liver.

## 2. Results

### 2.1. Asymmetrical Growth Restriction Was More Pronounced in Male Than Female Fetuses upon Systemic Human sFLT1 Overexpression at 18.5 dpc

***Fetal liver development and function upon systemic sFLT1 overexpression in pregnancy***

**Figure 1:**
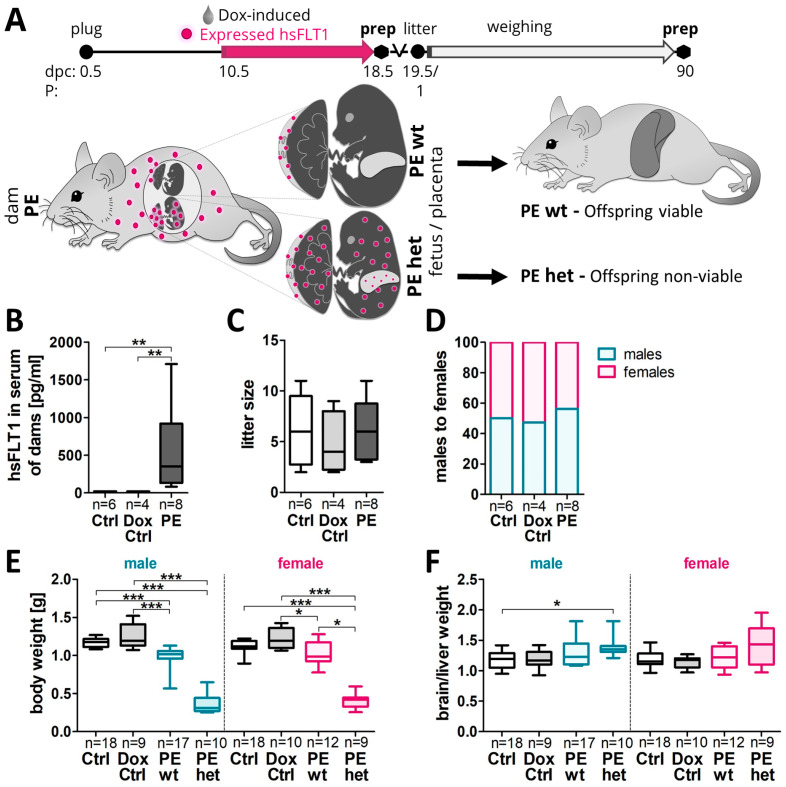
Systemic hsFLT1 overexpression resulted in reduced fetal body weight at 18.5 dpc. (**A**) In the experimental preeclampsia (PE) group, hsFLT1/rtTA females (homozygous for hsFLT1 and heterozygous for rtTA: hsFLT1+/+ and rtTA +/−) were mated with hsFLT1-transgenic males, lacking the rtTA allele (homozygous for hsFLT1 and wt for rtTA: hsFLT1+/+ and rtTA−/−). Thereby, one PE pregnancy included two types of fetuses in parallel: fetuses with the genotype hsFLT1+/+/rtTA+/− (PE het; heterozygous for rtTA) and fetuses with the genotype hsFLT1+/+/rtTA−/− (PE wt; wildtype for rtTA). Two control groups were performed. First with the same maternal genotype (hsFLT1+/+ and rtTA+/−) and the same mating strategy as for the PE group, named Ctrl. And second by mating single-transgenic hsFLT1 males and females lacking the rtTA allele (hsFLT1+/+ and rtTA−/−), which were used for doxycycline (Dox) side effects, named Dox Ctrl. At 10.5 until 18.5 dpc dams were either treated with Dox and sucrose (PE/Dox Ctrl) or with sucrose only (Ctrl). Consequently, in the PE dams, systemic hsFLT1 overexpression was induced from mid-gestation (10.5 dpc) until the end of the experiment (18.5 dpc). Since Dox passes the placental barrier, for fetal analyses, the maternal PE group was further subdivided into PE het fetuses (systemic maternal and feto-placental hsFLT1 overexpression) and PE wt fetuses (exclusive maternal hsFLT1 overexpression). The difference in the origin of hsFLT1 overexpression between PE wt (maternal) and PE het (maternal and fetoplacental) is illustrated with magenta dots. At the day of birth, Dox treatment was stopped, and the offspring weight gain was controlled until P90, i.e., young adulthood. Here, only the PE wt offspring are viable and could be analyzed. The sampling of fetal liver tissue was carried out at 18.5 dpc and the sampling of offspring liver tissue was carried out at P90. (**B**) hsFLT1 was exclusively present in the serum of induced PE dams with median levels about 351.7 pg/mL. (**C**) No differences were observed regarding the litter size. (**D**) In PE pregnancies the male fetuses (56.25%) seem to be more present than female fetuses (43.75%). (**E**) At 18.5 dpc, the fetal body weight was reduced in both types of hsFLT1 overexpression (PE wt and PE het) compared to both controls, with male fetuses more pronounced. (**F**) Moreover, especially male PE wt and PE het fetuses exhibited an increased brain/liver weight ratio. Data in (**B**,**C**,**E**,**F**) are partly shown in [20,21]. Data are presented as a box plot with the median, interquartile range ± upper/lower extreme; the sample size *n* of individual tested dams (**B**), litters (**C**,**D**) or fetuses (**E**,**F**) is listed under each graph. Statistics were conducted with Kruskal–Wallis combined with Dunn multiple comparisons test; * *p* < 0.05, ** *p* < 0.01 and *** *p* < 0.001.

In transgenic hsFLT1/rtTA (human sFLT1/reverse tetracycline-controlled transactivator) mice, hsFLT1 was systemically induced via Dox treatment in dams and fetuses or in dams only, depending on the fetal genotype. Of note, due to experimental design, PE dams carried both types of fetuses in parallel during one pregnancy. Fetuses having both transgenic alleles, hsFLT1 and rtTA, expressed hsFLT1 also by themselves (PE het), while fetuses lacking the rtTA allele did not express hsFLT1 (PE wt). The experimental set-up and the difference in the origin of hsFLT1 overexpression between the PE wt and PE het group is illustrated in Figure 1A. Whereas PE het fetuses were not viable, PE wt fetuses were viable and could be investigated postnatally. According to the experimental design, maternal analyses were performed for the whole litter (dams: PE, including PE wt and PE het), whereas fetal and placental analyses were further subdivided into two groups (fetus/placenta: PE subdivided into PE wt and PE het). At the end of the pregnancy, median maternal hsFLT1 serum levels were 351.7 pg/mL in PE dams (Figure 1B). No significant differences were seen in the litter size, but male fetuses (56.25%) were more present within a PE pregnancy than female fetuses (43.75%) (Figure 1C,D). At 18.5 dpc, fetuses of the PE group showed a reduction in body weight, which was even more pronounced in the PE het fetuses. Upon exclusive maternal hsFLT1 overexpression, i.e., PE wt, the reduced body weight was more evident in male than in female fetuses (Figure 1E). Upon hsFLT1 overexpression, the reduction in the total fetal body weight was further associated with an increase in the brain to liver weight ratio (as a marker for FGR) and male fetuses were more affected than females (Figure 1F). These data indicate that hsFLT1-induced PE resulted in different severity levels of asymmetrical growth restriction, in which viable male fetuses showed a stronger body weight reduction compared to their female siblings.

### 2.2. Fetal Livers Showed a Reduced Glycogen Storage Capacity and Increased Hemorrhages upon Systemic Human sFLT1 at 18.5 dpc

**Figure 2:**
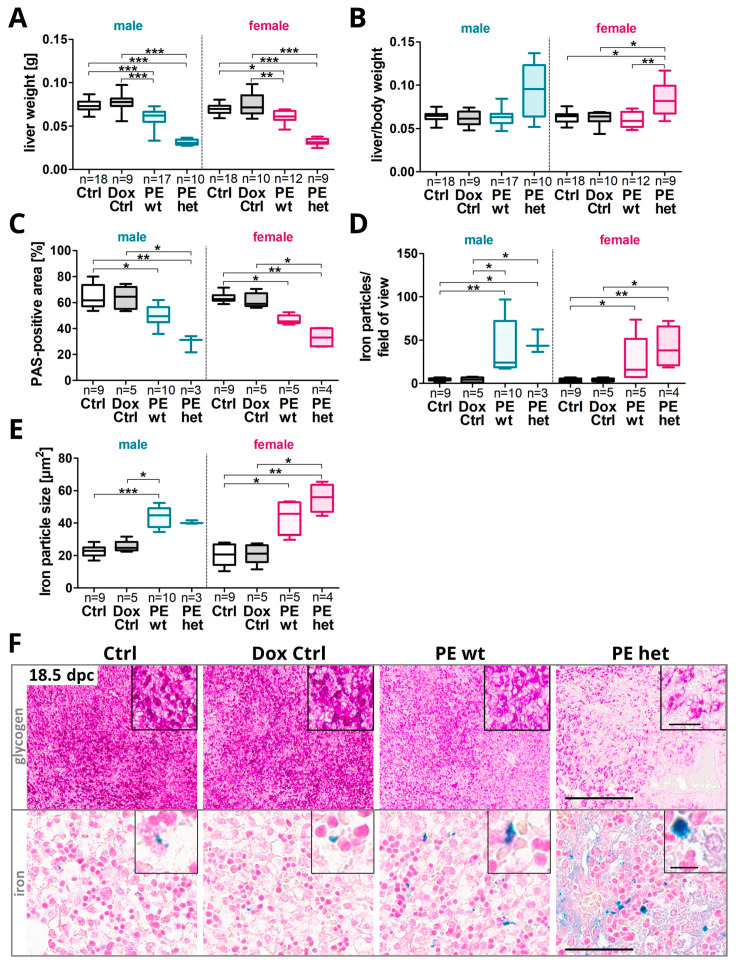
Reduced fetal liver glycogen storage and increased iron deposit presence upon systemic hsFLT1 overexpression at 18.5 dpc. (**A**) Fetal liver weight was reduced in both types of hsFLT1 overexpression in PE wt and PE het group, compared to controls (Ctrl and Dox Ctrl), (**B**) leading to increased liver to body weight ratio in PE het fetuses in both sexes. (**C**) Periodic Acid Schiff’s (PAS)-reaction was used to identify hepatic glycogen stores. The PAS-positive area of the liver was reduced upon hsFLT1 overexpression (PE wt and PE het), combined with (**D**) an increased number and (**E**) particle size of hepatic iron deposits detected by Perls Prussian Blue (Perls) reaction. Data are presented as the median, interquartile range and min/max. The sample size *n* is listed under each graph for the tested number of fetal livers per group. Statistics were conducted with Kruskal–Wallis and Dunn‘s multiple comparison post hoc test; * *p* < 0.05, ** *p* < 0.01 and *** *p* < 0.001. (**F**) Representative images of PAS (upper panel) and Perls reaction (lower panel) on transversal liver sections (scale bars: PAS staining: 300 μm overview; 50 µm insert; Perls: 60 μm overview; 10 µm insert).

It is known that offspring affected by preeclampsia may have an increased risk of obesity in adulthood. Since the fetal liver fulfils important metabolic functions including the storage of glycogen for energy supply and is acting as a hematopoietic organ, liver morphology was analyzed at the end of pregnancy (Figure 2A and Figure S1). As a human study suggested detrimental effects of PE combined with FGR regarding an association with a reduced number of erythropoietic cells within the fetal liver [25], this could be also linked to tissue hemorrhages and an increased number of iron particles. Thus, we investigated the amount of free iron in liver sections as a characteristic feature of hemorrhages.

Overall, investigating liver development and function, similar effects upon the systemic sFLT1 expression were seen in both sexes regarding the liver weight, liver/body weight ratio, the amount of iron deposits and glycogen storage. In detail: the fetal liver weight was reduced in both groups with hsFLT1 overexpression in PE wt and PE het fetuses compared to controls (Ctrl and Dox Ctrl), leading to an increased liver to body weight ratio in PE het fetuses in both sexes (Figure 2B). Only fetal PE het livers exhibited hsFLT1 overexpression by themselves (Figure S1A). No obvious differences were detectable in hematoxylin and eosin stainings regarding the presence of hepatoblasts and immature hepatocytes, erythroblasts or megakaryocytes, whereas hemorrhages (increased presence of erythrocytes) and ballooning (a histological sign of hepatocyte death) were exclusively present upon hsFLT1 overexpression in the organ and thereby only observed in PE het livers (Figure S1B). Inadequate placental angiogenesis during pregnancy can lead to placental hemorrhages or micro bleedings. Due to erythrocyte damage, Fe^2+^ can be released and oxidized to Fe^3+^ and thus detected by Perls reaction. We revealed an increased number and particle size of hepatic iron deposits in fetal livers upon hsFLT1 overexpression in both sexes (Figure 2D–F, lower panel). These results were associated with a reduced glycogen storage and increased iron particle number and size in the placentas of PE wt and het fetuses, most significantly in males (Figure S2). The PAS-positive area was reduced in PE wt and PE het livers, indicating reduced hepatic glycogen storage (Figure 2C,F, upper panel).

### 2.3. Systemic Human sFLT1 Leads to Altered Fetal Hepatic Gene Expression Metabolic Profiles at 18.5 dpc

Next, we investigated important hepatic nutrient transporters as well as key enzymes metabolizing fatty acids, glucose and glycogen, which are crucial factors for fetal growth and may be dysregulated in the fetal liver upon sFLT1-induced PE.

**Figure 3:**
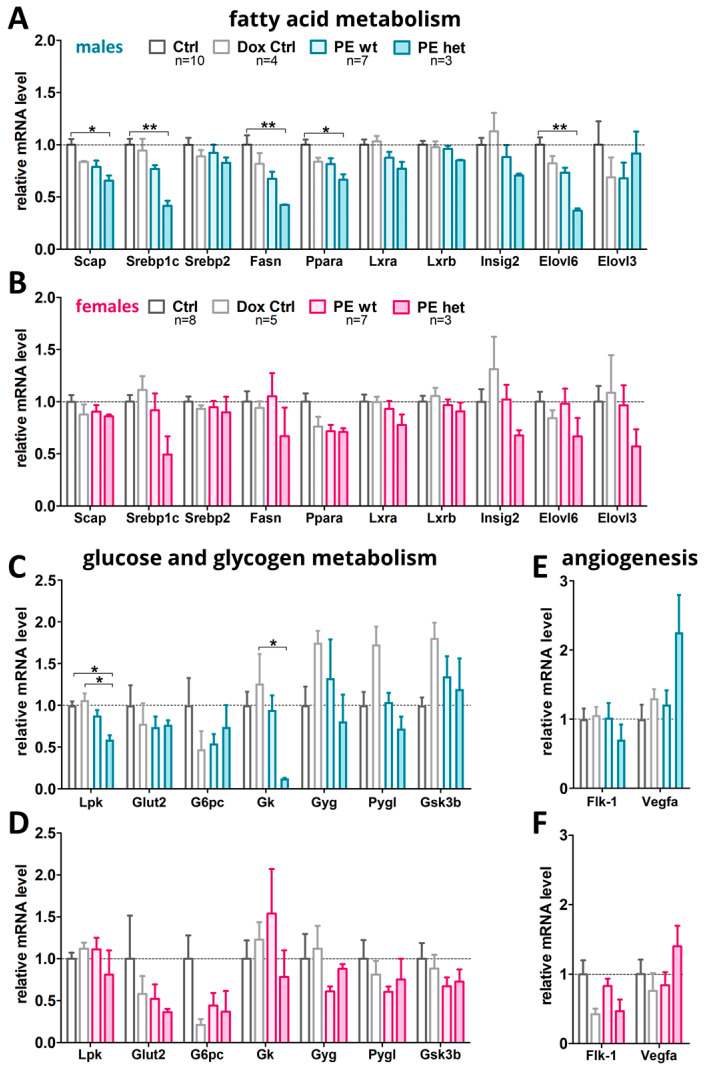
Marker gene expression in fetal liver tissue upon systemic hsFLT1 overexpression at 18.5 dpc. The following groups of fetal livers were analyzed: Ctrl, Dox-Ctrl and PE wt and PE het. (**A**,**B**) mRNA level of genes involved in fatty acid metabolism such as Srebp-cleavage-activating protein (Scap), sterol regulatory element-binding protein 1 and 2 (Srebp1, Srebp2), sterol regulatory element-binding transcription factor (Srebf), Fatty acid synthase (Fasn), peroxisome proliferator-activated receptor (Ppara), liver X receptor A and B (Lxra, Lxrb), Insulin-induced gene 2 (Insig2), Elongation of very long chain fatty acid-like 6 and 3 (Elovl6, Elovl3). (**C**,**D**) mRNA levels of members involved in glucose and glycogen metabolism such as liver pyruvate kinase (Lpk), Glucose-Transporter 2 (Glut-2), Glucose-6-phosphatase catalytic-subunit-C (G6pc), glycerol kinase (Gk), Glycogenin (Gyg), human liver glycogen phosphorylase (Pygl), Glycogen Synthase Kinase 3 β (Gsk3b). (**E**,**F**) mRNA levels of the Vegf-signaling pathway VEGF-receptor-2/fetal liver kinase 1 (Flk1), and vascular endothelial growth factor (Vegfa) as the angiogenesis marker. Data are presented as mean ± standard error of the mean normalized to 36b4 and Actinb (**A**,**B**) and glyceraldehyde-3-phosphate dehydrogenase (Gapdh) and β-Actin (Actb) (**C**–**F**) as housekeeping genes and final gene expression analysis was performed by ΔΔCT method. The sample size *n* for the tested fetal livers per group is listed in A and B for the graphs in (**A**–**F**). Statistics were carried out with Kruskal–Wallis and Dunn‘s multiple comparison post hoc test; * *p* < 0.05 and ** *p* < 0.01. Male livers (**A**,**C**,**E**), female livers (**B**,**D**,**F**).

For fatty acid metabolism, the most significant differences were found, which differed in both sexes and mainly affected males. mRNA levels of sterol regulatory element-binding protein cleavage-activating protein (*Scap*), sterol regulatory element-binding protein 1c (*Srebp1*), Fatty acid synthase (*Fasn*), peroxisome proliferator-activated receptor alpha (*Ppara*) and Elongation of very long chain fatty acid-like 6 (*Elovl6*) were significantly reduced in male PE het livers and reduced in male PE wt livers compared to controls, whereas in females no significant changes could be observed (Figure 3A,B). However, some genes also revealed a decrease in expression in PE het female livers such as Srep1c and Fasn.

Regarding the glucose and glycogen metabolism, significant changes were observed for liver pyruvate kinase (*Lpk*) and glycerol kinase (*Gk*), expressions of which are significantly reduced upon hsFLT1 overexpression, most severely in male PE het fetal livers (Figure 3C). Although not reaching statistical significance, in female livers most of the analyzed genes for glucose/glycogen metabolism also showed a decreased expression (Figure 3D). As important markers for angiogenesis, human sFLT1 (hsFLT1), VEGF receptor 1 (Flk-1) and Vegfa were analyzed. For both sexes, hsFLT1 is only expressed in PE het fetal livers (Figure S1A). Flk-1 expression is tended to be decreased upon hsFLT1 overexpression, more strongly in PE het fetal livers and Vegfa showed trends in increase (Figure 3E,F). In summary, a reduction in important fatty acid metabolic enzymes (*Srebp1, Fasn, Ppara*) and in two important genes for glucose metabolism (*Lpk*) and glycogen homeostasis (*Gk*) was observed upon the combined hsFLT1 overexpression in the dam and fetus.

### 2.4. Postnatal Weight Gain Was Increased in Male Adult Offspring after Preeclampsia-Affected Pregnancy

***Postnatal phenotype in adult offspring upon previous systemic sFLT1 overexpression regarding weight gain and liver metabolism***

**Figure 4:**
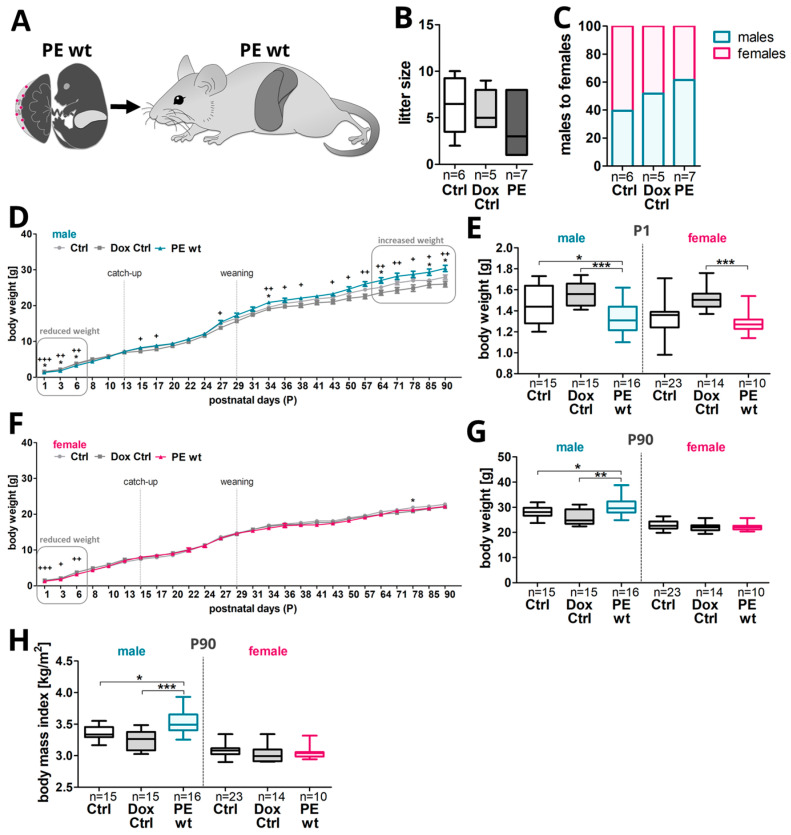
Offspring postnatal weight gain from the day of birth to an adult state, P90, upon previous human sFLT1-induced preeclampsia in mice. (**A**) The consequences of previous hsFLT1 upregulation during pregnancy on postnatal weight gain and the developmental progress of viable PE wt offspring. (**B**) The litter size was reduced in PE pregnancies after previous systemic hsFLT1 overexpression at the day of birth (postnatal day one [P1]). This was due to the fact that only PE wt offspring survived the pregnancy/birth and PE het fetuses died in utero or during birth. (**C**) As already seen at 18.5 dpc, the ratio between the male and female offspring shifted towards males in the PE group at the postnatal stage. (**D**) In males, the body weight was decreased in the first postnatal days, with a catch-up around P10–P13 in PE wt offspring with previous exclusive maternal systemic hsFLT1 overexpression. At P15 the weight increased for the first time, and since approximately P64 until the end of the experiment at P90, significantly higher than in the control male offspring (Ctrl and Dox Ctrl). (**E**) At the day of birth (P1), PE wt offspring showed a reduced body weight to both control groups (Ctrl/Dox Ctrl). Male PE wt offspring exhibited a stronger reduction than females. (**F**) Nevertheless, in females, PE wt offspring also started with a reduced body weight in the first postnatal days and caught up the weight around P13–15 but did not show an increased body weight afterwards. (**G**) At P90, PE wt males showed an increased body weight compared to controls (Ctrl and Dox Ctrl), combined with increased body mass index (BMI) (**H**). Data are presented as median, interquartile range and min/max or mean ± standard error of the mean. The sample size *n* is listed under each graph for the tested litters (**B**,**C**) and offspring (**D**–**H**) per group. Statistics were carried out with Kruskal–Wallis and Dunn‘s multiple comparison post hoc test; * *p* < 0.05, ** *p* < 0.01 and *** *p* < 0.001 and ^+^ *p* < 0.05, ^++^ *p* < 0.01 and ^+++^ *p* < 0.001 in (**D**,**E**) for comparison of Dox Ctrl to PE.

Regarding postnatal weight gain, a diagnostic parameter for obesity, body weight was measured 3 times a week from the day of birth, defined as postnatal day 1 (P1), until the postnatal day 90 (P90) (Figure 4A and Figure S3A). Even if not significant, the litter size in the PE group was reduced at the day of birth, due to the fact that PE het fetuses did not survive pregnancy or at least birth and only PE wt fetuses were viable (Figure 4B). Of note, no spontaneous offspring deaths after the day of birth were observed during the whole follow-up period. Further, the pups were raised by their corresponding dams. The weaning of the offspring was carried out as late as possible at P29 to keep the postnatal development of all pups similar. After weaning, the offspring were split into males and females with at least two male mice and at least three female mice in each cage. For each litter, the day on which each pup opened their eyes, as well as the day of an active and independent walking/movement of the pups were recorded as milestones in development. Both the day of eye opening as well as the day of active movement were delayed upon previous maternal systemic hsFLT1 overexpression during pregnancy (Figure S3C,E).

In males, in the first postnatal days (P1–P6), the body weight was lower in PE wt offspring compared to both control groups (Figure 4D,E), with a catch-up growth around P10–P13 (Figure 4D). At P15–P17 the body weight of PE wt males was increased for the first time, with peaks in body weight at P27, P34–P38, and from approximately P64 until the end of P90, the body weight was even higher in PE wt males than in male offspring of both control groups (Figure 4D,G).

In females, PE wt offspring also started with a reduced body weight in the first postnatal days (P1–P6) (Figure 4E). The highest catch-up regarding the total body weight was seen around P13–P15 in females, but afterwards no increased body weight was observed in PE wt females compared to controls (Figure 4F). In both sexes and all experimental groups, the highest percentual weight gain was observed from P3 to P6 with about a 75–80% of body weight increase compared to the previous day of measurement. An increased weight gain of hsFLT1-influenced offspring was seen in both sexes starting after P8 until P15, with a higher birth weight in male PE wt offspring compared to both control groups (Figure S3B) and in female PE wt offspring, the birth weight was only higher at P15 (Figure S3D). From P1 to P15, the Dox Ctrl group showed the highest body weight at birth, but the lowest weight gain in both sexes. A peak in percentual weight gain was seen in both sexes and for all experimental groups at P27, shortly prior to weaning, probably due to the beginning of eating normal pellet food besides lactation (Figure S3B,D). At P90 an increased absolute body weight and increased body mass index (BMI) in male PE wt offspring, a time point resembling young adulthood in mice, could be observed (Figure 4G,H).

### 2.5. Changes in the Hepatic Glycogen and Iron Presence in Adult Offspring after Preeclampsia-Affected Pregnancy

**Figure 5:**
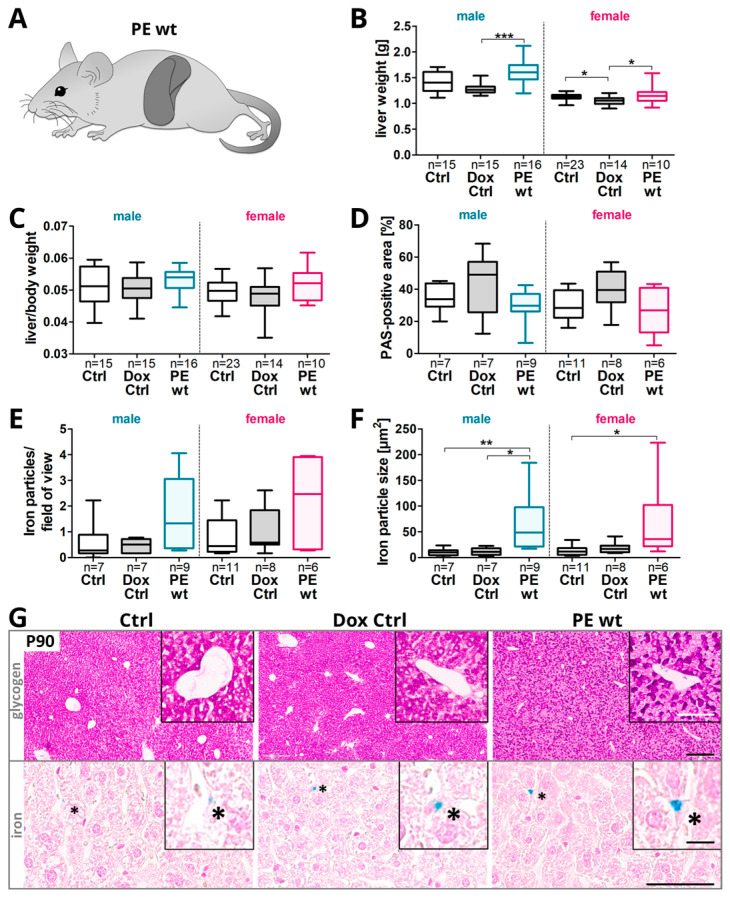
Offspring liver glycogen storage and iron deposit presence at P90 after the previous impact of systemic hsFLT1 overexpression during pregnancy. (**A**) The consequences of maternal hsFLT1 upregulation on adult offspring liver morphology. (**B**) At postnatal day 90 (P90), the offspring liver weight was increased in the PE wt offspring of both sexes, compared to controls (Ctrl and Dox Ctrl), combined with a tendency of an increased liver to body weight ratio especially in females (**C**). (**D**) Periodic Acid Schiff’s (PAS) reaction was used to identify the hepatic glycogen stores. The PAS-positive area of the liver tended to be reduced in PE wt offspring, combined with an increased number (**E**) and particle size (**F**) of the hepatic iron deposits detected by Perls Prussian Blue (Perls) reaction. Data are presented as median, interquartile range and min/max. The sample size *n* is listed under each graph for the tested livers per group. Statistics were carried out with Kruskal–Wallis and Dunn‘s multiple comparison post hoc test; * *p* < 0.05, ** *p* < 0.01 and *** *p* < 0.001. (**G**) Representative images of PAS (upper panel) and Perls reaction (lower panel) on transversal liver sections (scale bars: PAS: 300 μm overview; 100 μm insert; Perls: 60 μm overview; 10 μm insert).

Since adult P90 male offspring showed differences in postnatal weight gain, the liver morphology was analyzed (Figure 5A). At P90 the liver weight was increased in male PE wt offspring compared to both controls, with the lowest liver weight in the Dox Ctrl group. In females, the liver weight was decreased in the Dox Ctrl group compared to the Ctrl and PE wt groups (Figure 5B).

Nevertheless, in both sexes, the liver to body weight ratio additionally tended to be increased in PE wt offspring compared to both the controls (Figure 5C).

In both sexes, a slight reduction in the hepatic glycogen stores in the PE wt offspring was observed (Figure 5D,G, upper panel). This was further associated with an increased number and particle size of iron deposits (Figure 5E–G, lower panel).

### 2.6. Hepatic Metabolic Gene Expression Profiles in Adult Offspring after Preeclampsia-Affected Pregnancy

**Figure 6:**
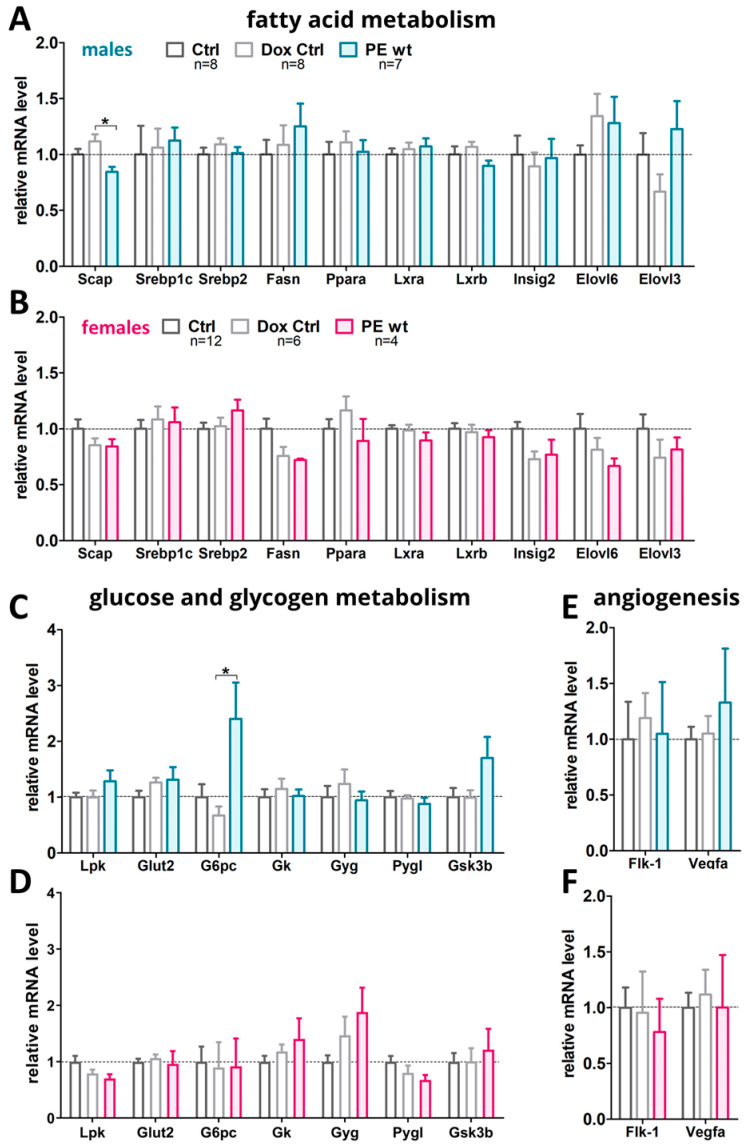
Marker gene expression in offspring adult liver tissue at P90 after the previous impact of systemic hsFLT1 overexpression during pregnancy. The following groups of adult livers were analyzed: Ctrl, Dox-Ctrl and PE wt. (**A**,**B**) mRNA level of genes involved in fatty acid metabolism such as Srebp-cleavage-activating protein (Scap), sterol regulatory element-binding protein 1 and 2 (Srebp1, Srebp2), sterol regulatory element-binding transcription factor (Srebf), Fatty acid synthase (Fasn), peroxisome proliferator-activated receptor (Ppara), liver X receptor A and B (Lxra, Lxrb), Insulin-induced gene 2 (Insig2), Elongation of very long chain fatty acid-like 6 and 3 (Elovl6, Elovl3). (**C**,**D**) mRNA levels of members involved in glucose and glycogen metabolism such as liver pyruvate kinase (Lpk), Glucose-Transporter 2 (Glut2), Glucose-6-phosphatase catalytic-subunit-C (G6pc), glycerol kinase (Gk), Glycogenin (Gyg), human liver glycogen phosphorylase (Pygl), Glycogen Synthase Kinase 3 β (Gsk3b). (**E**,**F**) mRNA levels of the Vegf-signaling pathway VEGF-receptor-2/fetal liver kinase 1 (Flk-1) and vascular endothelial growth factor (Vegfa) as the angiogenesis marker. Data are presented as mean ± standard error of the mean normalized to 36b4 and Actinb (**A**,**B**) and glyceraldehyde-3-phosphate dehydrogenase (Gapdh) and β-Actin (Actb) (**C**–**F**) as housekeeping genes and final gene expression analysis was performed by ΔΔCT method. Sample size *n* for the tested offspring livers per group is listed in A and B for graphs in (**C**–**F**). Statistics were carried out with Kruskal–Wallis and Dunn‘s multiple comparison post hoc test; * *p* < 0.05. Male livers (**A**,**C**,**E**), female livers (**B**,**D**,**F**).

Since the morphological analysis of the adult livers did not show any severe difference in the PE wt offspring, the important genes for the fatty acid metabolism and glucose/glycogen metabolism were investigated (Figure 6). Additionally, the crucial factors for angiogenesis were analyzed. Only the male offspring revealed some significant differences in liver gene expression.

*Scap* was reduced in male PE wt livers compared to the controls (Figure 6A). In female livers, no such alteration in *Scap* or any other investigated gene was detected (Figure 6B). For the glucose/glycogen metabolism, the most obvious changes were detected in the male adult livers of the PE wt offspring with a significant increase in *G6pc* as well as trends in increase in *Lpk*, *Glut-2* and *Gsk3b* (Figure 6C). In female livers, no differences between the experimental groups were found (Figure 6D). The angiogenic genes were not influenced by the maternal hsFLT1 overexpression (Figure 6E,F). hsFLT1 mRNA was not expressed in any of the experimental groups.

### 2.7. Increased Offspring Postnatal Weight Gain in Males Was Associated with an Increase in Serum Hormones, Cytokines and Triglyceride Levels

**Figure 7:**
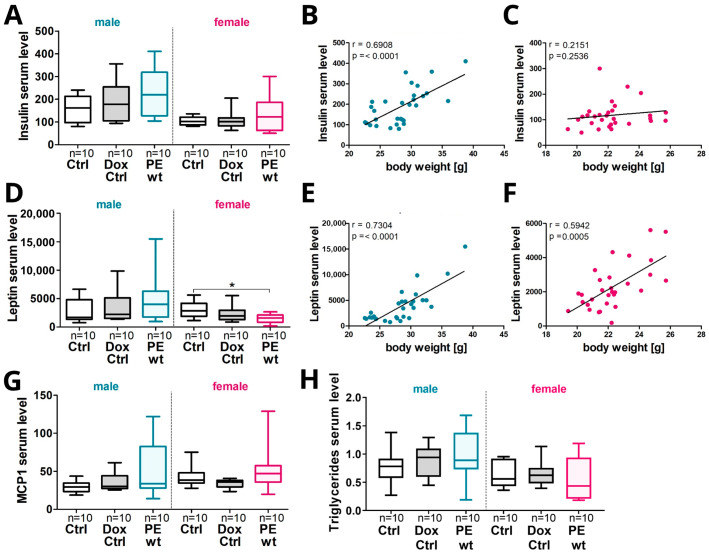
Increased offspring postnatal weight gain in males at P90 correlated with serum Insulin and Leptin levels in previously growth restricted offspring after the exposure to systemic maternal hsFLT1 overexpression during pregnancy. (**A**) The Insulin serum level is increased in the male PE wt offspring with previous exclusive maternal systemic hsFLT1 overexpression, compared to controls (Ctrl and Dox Ctrl). (**B**) In males, an increase in serum Insulin positively correlated to an increase in body weight, whereas this was not the case in females. (**C**,**D**) The Leptin serum level is increased in the male and decreased in the female PE wt offspring compared to controls (Ctrl and Dox Ctrl). (**E**,**F**) In both sexes, an increase in serum Leptin positively correlated to an increase in the body weight. (**G**) The MCP-1 serum level is increased in males and the female PE wt offspring. (**H**) The triglyceride levels are increased in male PE offspring compared to controls and females. Data are presented as median, interquartile range and min/max. The sample size *n* is listed under each graph for the tested number of individual serum samples per group. Statistics were carried out with Kruskal–Wallis and Dunn‘s multiple comparison post hoc test; * *p* < 0.05 and Spearman correlation (**B**,**C**,**E**,**F**).

Since the male PE offspring showed an increased postnatal weight gain, a subset of metabolic active obesity-related hormones, such as Ghrelin, Glucagon, Insulin, Leptin and PYY, were analyzed. In addition, the triglyceride levels as well as the cytokines Il-6, MCP-1 and TNFα were investigated in the serum. At P90, the offspring serum Insulin levels tended to be increased in PE wt males compared to controls. In males, an increase in the serum Insulin positively correlated to an increase in body weight, whereas this was not the case in females (Figure 7A–C). Furthermore, the offspring serum Leptin levels tended to be increased in PE wt males and significantly decreased in PE wt females. For both sexes, the increase in serum Leptin positively correlated to the body weight (Figure 7D–F). Additionally, the serum MCP-1 levels tended to be increased in male and female PE offspring compared to controls, but without a correlation to the body weight (Figure 7G, correlation not shown). Serum levels of Ghrelin, Glucagon, IL-6, PYY and TNFα did not differ in the offspring between the experimental groups or between the sexes. Additionally, triglycerides were elevated in the PE wt offspring in males but not in females (Figure 7H) without a significant correlation to the body weight.

## 3. Discussion

In this study, we demonstrate that the systemic overexpression of human sFLT1 in mice, which is one of the main drivers of human preeclampsia, not only altered liver morphology and function in the fetus, but also had postnatal effects in the offspring, affecting males more than females. This resulted in a changed metabolic phenotype in the offspring, which is strongly associated with obesity.

### 3.1. Systemic sFLT1 Overexpression during Pregnancy in Mice Leads to a Changed Pathophysiology in the Fetal Liver

We investigated the liver development and function including the glycogen storage capacity in more detail in the fetus. Combined with a reduced liver weight at term, reduced glycogen-enriched areas were observed in the livers upon hsFLT1 overexpression in PE mice. This was in line with our results of previously described impaired placental phenotype of these mice [15] exhibiting a reduced nutrient transport across the placenta and reduced nutrient availability in the FGR fetuses. In this previous study, we showed a strong effect of systemic hsFLT1 overexpression during pregnancy on placental nutrient transporter expression, characterized by a reduced expression of several transporters, such as glucose transporter 1 and 3 (*Glut-1*; *Glut-3*), amino acid transporters, solute carrier family 38 member one and two (*Slc38a1*; *Slc38a2*), and most severely the fatty acid translocase *Cd36* and fatty acid binding protein 3 (*Fabp3*). This was associated with an accumulation of phospholipids detected in the maternal serum. Previously, we already revealed a reduced placental glycogen storage at 18.5 dpc by using a mouse model with lentiviral hsFLT1 overexpression in blastocysts [14]. The reduced glycogen stores in the fetal PE livers as well as the reduced liver expression of important signaling enzymes in glucose metabolism, such as liver pyruvate kinase (*Lpk)* and glycerol kinase (*Gk),* are in accordance with our previous study of the transgenic hsFLT1 mice. There, we revealed a reduced placental exchange area of the labyrinth and glycogen stores and a decreased expression of glucose transporters [15], concluding that systemic hsFLT1 expression resulted in an impaired intrauterine availability of nutrients leading to FGR. These findings also fit to the often-observed FGR phenotype in human PE, which is characterized by placental dysfunction leading to a reduced nutrient supply to the fetus and thus FGR.

Generally, the glycolytic enzymes LPK and GK are found to be decreased during fasting [26]. In our PE mice study, we see similar results without prior fasting of the dams, indicating a changed response towards the hunger signals in these offspring born under PE conditions. However, the in vivo measurement of serum glucose and Insulin levels has to be performed in the future to assess the glycemic status of the offspring suffering from previous PE pregnancies. In PLGF-KO mice, the placental compartment is characterized by enlargement of the junctional zone due to glycogen accumulation and a reduction in the labyrinthine area [27]. This increase in the glycogen stores is discussed as a form of protection and counter-regulation against the increased disturbances in feto-placental metabolism, which might be a PLGF-specific effect. Sato et al. [28] confirmed our results and revealed in mice administered with recombinant sFLT1 adenovirus at 8.5 dpc that plasma and placental acylcarnitine levels were significantly higher in PE mice than in control mice. Glycolysis was accelerated and the glucose metabolic flow was altered with hypoxia, leading to an ATP shortage in the labyrinth of PE mice [28]. In these PE mice, ATP production was diminished and fatty acid oxidation was accelerated in the placenta. Consequently, the blood carnitine and acylcarnitine levels were increased.

In this study we detected changes in the regulation of the hepatic fat metabolism in PE mice. As previously shown in a study by Stojanovska et al. [29], in utero sFLT1 exposure affected fatty acid metabolism genes and the *Ppara* targets in fetal rat livers. However, these rats overexpressing sFLT1 did not develop hypertension or proteinuria, two main symptoms of PE, which were seen in our sFLT1 transgenic mice. In the present study, we found several differences in gene expression for *Scap*, *Srebp1c*, *Fasn*, *Ppara* and *Elovl6* in 18.5-day-old fetal mouse livers. SREBP is the critical protein during sterol regulation [30]. As the key regulator of SREBP maturation, we found a downregulation of the *Scap* gene in PE livers, together with *Srebp1c*. SREBPs play important roles in regulating fatty acid biosynthesis and they also activate the downstream genes involved in the fatty acid biosynthesis [31]. The expression of the *Fasn* gene, which encodes for fatty acid synthetase, was also declined as it is controlled by *Lxrs* and *Srebp1* [32]. PPARA prevents hepatic lipid storage [33]. A former study showed that the protein expression of hepatic PPARA was decreased in intrauterine growth-restricted newborn rats [34], which is consistent with our finding showing a decreased expression of *Ppara* gene in PE fetuses. ELOVL6 regulates lipogenesis and fatty acid oxidation and in *Elovl6^−/−^* mice decreased SREBP-1c and PPARα expression was observed [35]. In this study, we found a reduced *Elovl6* gene expression in the liver of PE het fetuses. Interestingly, a study investigating the acute fatty liver disease in human pregnancy showed that this disease is associated with elevated maternal serum levels of sFLT1, even higher, as in early preeclamptic patients [36]. This suggests a potential role of sFLT1 in the liver pathophysiology characterized by an increased lipid storage. Our results revealed only significantly reduced fetal hepatic expression levels in fatty acid metabolism in males rather than in females. In a study by Stojanovska et al. [17], mRNA level of *Srebp1, Lxr* and *Fasn* also tended to be decreased in fetal male livers in a mouse model for FGR without PE. In this mouse model, the major trophoblast differentiation regulator transcription factor AP-2y (*Tfap2c*) was genetically deleted, which induced a placental insufficiency and FGR at term, but without typical PE symptoms in the dam. This could be a hint that the observed changes in the liver fatty acid metabolism upon hsFLT1 overexpression are linked to FGR and not to PE itself. As discussed above, unexpectedly the various gene expression differences as well as the reduced glycogen capacity found in the fetuses are not present anymore at P90, except for small differences, such as seen for *Scap*.

In a recent human metabolomic analysis of 30 healthy pregnant women and 26 patients suffering from PE, sFLT1, sFLT1/PlGF ratio and sENG levels showed a positive correlation with triglyceride, free fatty acid and C-peptide maternal serum levels [37]. This highly supported our findings that increased levels of anti-angiogenic sFLT1 resulted in early maternal metabolic changes, also influencing fetal metabolism [15]. As reviewed in [38], neonates born from PE mothers showed increased levels of lipoproteins and also oxidative damage in lipoproteins [39].

During fetal development, the liver is an additional hematopoietic tissue, which produces blood stem cells prior to bone marrow development [24]. Thus, defects in liver function during fetal development can have severe consequences for the fetal well-being during the pregnancy and can have further metabolic or hematopoietic consequences for the offspring. In this study, sFLT1 overexpression resulted in an increased amount of iron deposits in fetal livers and placentas, indicating an increase in hemorrhages and apoptosis. It is known that genetic deletion of Vegf in mice induces endothelial cell apoptosis, leading to abnormal vessel development, including hemorrhages in several tissues as well as fetal lethality [40]. In humans, an increase in the iron deposit presence in trophoblasts is associated with fetal abnormalities and PE [41], which might be a consequence of non-hemorrhagic metabolic events, such as hypoxia and oxidative stress often observed in PE. This could also affect the hematopoietic system of the liver during fetal development. In a human study, an impact of PE combined with FGR on the number of erythropoietic cells within the fetal liver was found [25]. Extramedullary hematopoiesis in the fetal liver revealed an increased apoptosis of erythroid blasts upon PE/FGR. These findings further support the hypothesis that hsFLT1 overexpression during pregnancy and PE itself not only has an influence on vascularization or nutrient and oxygen supply to the placenta and fetus, but might also have a negative influence on erythropoiesis during pregnancy as observed in the sFLT1 mice.

### 3.2. Systemic sFLT1 Overexpression during Pregnancy in Mice Leads to Increased Postnatal Weight Gain in the Adult Male Offspring

The strongest influence on offspring development upon previous hsFLT1 overexpression during pregnancy was found in the context of postnatal weight gain and body weight regulation. Here, male PE offspring showed an increased postnatal weight gain and body weight in adulthood, combined with an increase in serum Insulin and Leptin levels, which positively correlated to the body weight. For Leptin this was expected, since this hormone is primarily produced by the adipose tissue in proportion to the size of fat stores [42]. These findings could be first signs of developing obesity, metabolic syndrome or a beginning of Insulin resistance, and therefore later on type 2 diabetes as shown in previous growth-restricted children of PE pregnancies, which have a higher risk of developing metabolic diseases or obesity [22]. Here, the male preterm infants of PE pregnancies seem to be more affected, having an increased BMI in comparison to premature males born from normotensive pregnancies. The metabolic responses upon experimental PE with an increased weight gain and changes in serum metabolic or hormonal profiles are common features following FGR during pregnancy. This could be linked to a reduced bioavailability of nutrients during the fetal development and thus an adaptation of the fetus to the undersupply. As shown in this study, this negative preprogramming of the fetus could be further impaired upon superimposed hsFLT1 overexpression combined with maternal PE syndrome during pregnancy. Thus, our results found in the sFLT1 mouse model could be linked to the findings observed in the clinic.

Additionally, influences on the offspring metabolic phenotype in a sex-specific manner could be observed in many PE animal models [43,44]. The undernutrition of the fetuses during pregnancy led to adiposity in adulthood, combined with increased plasma glucose, triglycerides and corticosterone levels, as well as increased hepatic cholesterol and triglyceride levels in male offspring. Moreover, hypoxia during pregnancy in mice led to an increase in adipocyte size, increased plasma Insulin, Leptin, triglyceride and free fatty acid levels, combined with reduced Insulin sensitivity [45]. In the rat RUPP (reduced uterine perfusion pressure) PE model mediated by bilateral uterine vessel ligation Hassanzadeh-Taheri et al. [46] also revealed in male offspring (P60) a significantly higher body weight and a decrease in Insulin levels in young adult rats after previous PE. It has already been reported that pups born after preeclampsia and FGR compensate for their growth restriction in the postnatal period [47].

It is known that males are more likely to develop obesity, Insulin resistance and hyperglycemia than females [48]. As an explanation for the sex differences, steroid hormone levels such as endogenous estrogens are discussed as protective in various tissues, including the brain, the liver and adipose tissue. However, which underlying mechanisms and factors contribute to the metabolic response are unknown so far and need to be further investigated. Further, it is well known that placental and fetal responses to nutrient supply frequently show sexual dimorphism [49]. In mice, where PE was induced by a combined exposure to sFLT1 and Lipopolysaccharides (LPS), growth-restricted male fetuses showed significant changes in their amino acid metabolism, as well as a brain-sparing effect [16]. These effects could not be detected in females, which led to the conclusion that females may have greater ability to adapt to fluctuating conditions compared to males [50]. Moreover, in aged female mice in a FGR mouse model, placental insufficiency may lead to higher lipogenesis, at least indicated by greater *Fasn* expression in liver and white adipose tissues [17]. In human, Bi et al. [7] summarized the long-term effects of PE in a meta-analysis on metabolic outcomes. This analysis revealed that children of PE mothers had an increased risk of obesity in childhood and a higher body mass index and blood pressure in puberty, but no differences in the blood lipids or glucose metabolism compared to non-PE offspring. Thus, PE might be associated with a higher risk for central obesity, hypertension and type 2 diabetes mellitus of offspring in later life [7].

Overall, our study might reveal new insights into fetal and postnatal metabolic responses during FGR and PE upon hsFLT1 exposure. One possible cause of sex-specific differences in the offspring could be due to epigenetic changes during fetal development. Many of the genes studied here are well-known as targets of differential methylation, e.g., the PPARs and LXRs [51,52]. To gain a better understanding of the sex-specific differences in fetal and offspring programming mechanisms upon hsFLT1 upregulation in our established transgenic PE mouse models, the epigenetic changes should also be considered and further analyzed in the future.

In conclusion, we presented data of an altered fetal and offspring metabolic phenotype in a clinically relevant hsFLT1 mouse model that closely mimics human preeclampsia. Here, the systemic overexpression of human sFLT1 in pregnant mice resulted in various sex-specific differences in fetal and adult liver development accompanied by an altered growth restriction pattern. Additionally, the offspring’s serum hormones were affected by sFLT1 overexpression shown by the changed metabolomic responses mostly affecting males.

## 4. Materials and Methods

### 4.1. Animals

The detailed information for the mouse line generation and the underlying genetics of hsFLT1/rtTA mice has been previously published [15,20]. For details of the generation of the hsFLT1/rtTA mouse and the experimental set-up, see [15]. Shortly, mice harboring the hsFLT1 gene were generated on a 129/Sv background and the allele was registered in the mouse genome informatics database; named Col1A1tm2 (tetO−Flt1*) Hsc (MGI: 6202353). These mice were mated with Rosa26–rtTA-M2 mice (B6.Gt (ROSA)26Sortm1 (rtTA*M2)Jae, Stock No. 006965; Jackson Laboratory, Bar Harbor, ME, USA) to generate hsFLT1/rtTA double transgenic mice.

The experimental PE group is defined by double-transgenic dams having both transgenic alleles (hsFLT1 and rtTA) and receiving 2 mg/mL doxycycline (Dox) (0.2% [*w*/*v*]; Merck, Darmstadt, Germany) and 30 mg/mL sucrose (3% [*w*/*v*]; Carl Roth, Karlsruhe, Germany) in the drinking water to induce systemic hsFLT1 overexpression from mid-pregnancy (10.5 dpc) until nearly the end of pregnancy (18.5 dpc). The control group (Ctrl) is defined by double-transgenic dams (hsFLT1 and rtTA), like dams of the PE group, but receiving only 30 mg/mL sucrose in the drinking water without Dox. Thus, the expression of hsFLT1 is not possible. Further, a second control group was used to test for Dox’s side effects (Dox Ctrl). The Dox Ctrl group is defined by single-transgenic hsFLT1 mice lacking the rtTA allele and received, like the PE group, 2 mg/mL Dox and 30 mg/mL sucrose in the drinking water, not expressing hsFLT1. Since Dox passes the placental barrier, double transgenic hsFLT1/rtTA fetuses/placentas (PE het: homozygous for hsFLT1 and heterozygous for rtTA) can also express hsFLT1, whereas single transgenic fetuses/placentas (PE wt: lacking the rtTA allele) cannot. Table 1 displays the differences between the maternal and fetal experimental groups. Figure 1A illustrates the mating strategy and distribution of hsFLT1 expression in PE wt fetuses (exclusive maternal) compared to PE het group (maternal and feto-placental). The sampling of placentas and fetuses was performed at 18.5 dpc on the total number of dams (Ctrl *n* = 6, Dox Ctrl *n* = 4, PE *n* = 8). Each dam only gave birth to one litter and the litter size were standardized to 4–7 pups for each sex to ensure no litter was nutritionally biased. By natural circumstances, the litter size of some dams was reduced further. This was due to the fact that only PE wt offspring survive. In addition, for each litter the amount of fetus/placenta was subdivided and collected in a 50:50 ratio for histological as well as molecular biological analysis. For the analysis of the offspring at P90, a total number of dams (Ctrl *n* = 6, Dox Ctrl *n* = 5, PE *n* = 7) was used. From each litter 1–5 offspring per sex were used for the analysis. The exact number of dams, placentas, fetuses and offspring per litter is given below for each experiment separately. Mice were housed in a specific-pathogen–free environment at the animal facility of the University Hospital Essen, were exposed to cycles of 12 h of light/dark and were provided with food and water ad libitum. All animal procedures were performed in accordance with the German laws for animal protection (No.: G1644/17).

### 4.2. Body Mass Index Calculation

The body mass index was calculated to receive a comparable parameter to analyze both changes of the body weight and body length/body surface area. The body surface area was derived from the DuBois equation:

$$body\;surface\;area\;\left[m^{2}\right]=0.007184*(weight\;{\left[\mathrm{kg}\right]}^{0.425})*(lenght\;{\left[\mathrm{cm}\right]}^{0.725})$$

The mouse body length was measured from the tip of the nose to the base of the tail in cm. The body mass index (BMI) was calculated as follows:

$$BMI=\left[\frac{\mathrm{kg}}{m^{2}}\right]\frac{body\;weight\;\left[\mathrm{kg}\right]}{body\;surface\;area\;\left[m^{2}\right]}$$

### 4.3. Tissue Preparation

Tissue preparation was carried out as previously described [15,20,21]. At 18.5 dpc the pregnant mice were anesthetized via a mixture of air and 5% [*v*/*v*] Isoflurane and killed by cervical dislocation. The maternal blood was collected by cardiopuncture and fetuses were isolated and weighed with an ALJ 220–4NM analytical balance (Kern, Ebingen, Germany) with a linearity of ±0.2 mg. Afterwards, the fetuses were dissected in ice cold sterile phosphate-buffered saline (PBS) and the fetal organs were isolated and weighed. The organs were either frozen and stored at −80 °C (for RNA, DNA) or immediately fixed in ice cold 4% [*w*/*v*] paraformaldehyde (PFA) for 24 h at 4 °C and stored in 70% [*v*/*v*] ethanol at 4 °C until being embedded in paraffin standard procedures (for morphology).

### 4.4. Serum hsFLT1 Measurements via Brahms Kryptor

Serum samples of dams were prepared as previously described [15,20,21]. Briefly, whole blood samples were coagulated for 2 h at room temperature. After centrifuging the clotted blood for 15 min at 3000× *g* and 4 °C, the undiluted serum sample was used to measure the concentration of hsFLT1 with a BRAHMS KRYPTOR compact PLUS analyzer (Brahms GmbH, Henningsdorf, Germany), according to the manufacturer’s protocol.

### 4.5. Serum Hormone, Cytokine, and Triglyceride Measurements

Next, 17 µL of the undiluted serum sample of P90 offspring was used to measure the concentration of Ghrelin, Glucagon, Interleukin-6 (IL-6), Insulin, Leptin, Monocyte-chemoattractant protein 1 (MCP-1), Peptide YY (PYY) and Tumor necrosis factor-alpha (TNFα) with a Meso Scale analyzer and the U-PLEX Metabolic Hormones Combo 1 Kit (MSD, Rockville, MD, USA), according to the manufacturer’s protocol. The samples were diluted 1:3 in the Metabolic Assay Working Solution provided in the kit. The detection levels ranged from 1.7 to 2710 pg/mL for Ghrelin, from 0.13 to 156 pM for Glucagon, from 3.0 to 5500 μIU/mL for Insulin, from 11 to 50,000 pg/mL for Leptin, from 1.4 to 1400 pg/mL for MCP-1, from 4.8 to 16,000 pg/mL for IL-6, from 1.1 to 4000 pg/mL for PYY and from 1.3 to 6200 pg/mL for TNFα. The results were analyzed using the MSD DISCOVERY WORKBENCH 4.0. analysis software. The serum triglycerides were measured via a calorimetric enzymatic assay according to the manufacturer’s instructions (DiaSys, Holzheim, Germany).

### 4.6. Hematoxylin and Eosin Staining

For the general liver morphology, a hematoxylin and eosin (HE) stain was used as previously described [15,20,21].

### 4.7. Periodic Acid Schiff’s Reaction

Glycogen cells are recognized histochemically as containing glycoprotein-rich, cytoplasmic granules that react with Periodic Acid Schiff’s (PAS) reagent. For the histological visualization of glycogen in the fetal and postnatal livers, PAS reaction was used, which was detailed described previously [20]. The PAS-positive area was calculated in liver sections (nine images each sample) as well as single snapshots of liver sections (18 images each sample).

### 4.8. Perls Prussian Blue Reaction

An inadequate placental angiogenesis during pregnancy can lead to placental hemorrhages or micro bleedings. The Perls Prussian Blue reaction detects and identifies free ferric (Fe^3+^). Normally, Fe^3+^ is stable bound in form of Fe^2+^ to iron-storing proteins, for example the hemoglobin of erythrocytes. Due to erythrocyte damage, Fe^2+^ can be released and oxidated to Fe^3+^, and thus, detected by Perls reaction.

Perls Prussian Blue (Perls) Iron Special Stain Kit (38016SS7, Leica Biosystems, Wetzlar, Germany) was used according to the manufacturer’s protocol as already performed in [21]. Briefly, 4% ferrocyanide solution and 4% acidified water were mixed immediately before incubating the slides for 45 min then counter-stained with 1% neutral Red. The number and size of Perls-positive particles were calculated (nine images each sample).

### 4.9. Quantitative Image Analysis

For all histological analyses, formalin-fixed and paraffin-embedded samples were used. For fetal and adult liver analyses, the livers were sectioned transversal at 5 µm and mounted on Superfrost Plus Slides (R. Langenbrinck, Emmendingen, Germany). The stained slides were scanned with the Aperio CS2 ScanScope slide scanner (Leica, Wetzlar, Germany) at 20× or 40× magnification (Westdeutsche Biobank, University Hospital Essen, Essen, Germany) and images were exported as TIFF via Image Scope (Version 12.3.2.8013; Leica). Exported slides were opened (plugin “bioformats_package.jar.”) and analyzed using Fiji/ImageJ [53]. For the quantitative image analysis of special stains (Perls and PAS), the original exported Red–Green–Blue (RGB) image was split into an 8-bit image containing exclusively the information of one color of each staining: Perls Prussian Blue (blue) or Periodic Acid Schiff (magenta). Afterwards, each image was thresholded to find a binary black and white image, showing in white the staining of interest and in black the background. By using the “watershed” algorithm single iron particles (recognized by Perls) were separated, and the number and size were calculated with the “analyze particle” command. To calculate the glycogen containing area (recognized by PAS), the percentage of stained (white area) compared to unstained background (black area) was calculated.

### 4.10. Genomic DNA Isolation, Genotyping and Sex Determination

Genomic DNA was isolated from ear punch, or fetal tail tissue samples with the REDExtract-N-AmpTM Tissue PCR Kit (#XNAT; Sigma-Aldrich, St. Louis, MO, USA) according to the manufacturer’s protocol. Genotyping and sex determination of mice were performed as previously described [15,20,21] and the appropriate primers listed in Table S4.

### 4.11. RNA Extraction, cDNA Synthesis and Quantitative Polymerase Chain Reaction

Total RNA from ~20 mg fetal or adult mouse liver was extracted by using the AllPrep DNA/RNA Mini kit (Qiagen, Germany).

For the analysis of angiogenesis, glucose/glycogen metabolism and glycogen homeostasis, cDNA synthesis and quantitative polymerase chain reaction was performed as previously described [15,20,21]. The gene expression was measured from 1 μL cDNA with 19 μL of the PowerUP SYBR Green Master Mix (#A25742; Applied Biosystems, Foster City, CA, USA) and the ABI Prism 7300 Sequence Detection System (Applied Biosystems, Waltham, MA, USA). The amount of cDNA in each sample was normalized to the mean level of glyceraldehyde-3-phosphate dehydrogenase (Gapdh) and β-Actin as the housekeeping genes and the final gene expression analysis was carried out by the ∆∆CT method.

For the analysis of the fatty acid metabolism, 1–2 µg of total RNA was converted into first-strand cDNA using RNaseOUT (Invitrogen, Waltham, MA, USA), M-MLV reverse transcriptase (Invitrogen, Waltham, MA, USA) and random nonamer primers (Sigma-Aldrich, St. Louis, MO, USA). Taqman Fast Advanced Master Mix (Applied Biosystems, Waltham, MA, USA) was used for real-time qPCR and all the qPCR runs were performed in triplicate in 96-well plates on Quantstudio 3 hardware (Applied Biosystems, Waltham, MA, USA). Relative mRNA expression was determined by the standard curves, normalized to the mean level of Actinb-1 and 36b4 as the housekeeping genes. All primer sequences are listed in Supplementary Table S1.

### 4.12. Statistics

The normal distribution was tested with D’Agostino-Pearson omnibus K2 test and Shapiro–Wilk test, which could not prove the Gaussian distribution for all data sets. Therefore, the statistics was performed with Kruskal–Wallis and Dunn’s multiple comparison test to compare three or more experimental groups. Data are presented as box plots, with median, interquartile range ± minimum to maximum; or mean ± standard error of the mean (SEM). Individual sample size (*n*) is listed under each graph. A probability value (*p* value) of 0.05 or less was indicated with *, ** *p* < 0.01 and *** *p* < 0.001. Data were analyzed with GraphPad Prism software version 5.01 (GraphPad, La Jolla, CA, USA). No outliers were detected by performing Grubbs’ test (https://www.graphpad.com/quickcalcs/Grubbs1.cfm (accessed on 10 November 2022)).

## Figures and Tables

**Table 1:** Maternal and fetal experimental groups.

Maternal Groups		Fetal Groups	
Ctrl Genotype: hsFLT1/rtTA	No hsFLT1 expression, without Dox treatment	Ctrl Genotype: hsFLT1/rtTA	No hsFLT1 expression
Dox Ctrl Genotype: hsFLT1	No hsFLT1 expression, with Dox treatment	Dox Ctrl Genotype: hsFLT1	No hsFLT1 expression
PE Genoype: hsFLT1/rtTA	Systemic hsFLT1 overexpression, with Dox treatment	PE wt Genotype: hsFLT1 PE het Genotype: hsFLT1/rtTA	Exclusive maternal systemic hsFLT1 overexpression Combined maternal and fetal systemic hsFLT1 overexpression

## Data Availability

The authors declare that all supporting methods are available within the article. The underlying data that support the findings of this study are available from the corresponding author on reasonable request.

## Supplementary Materials

The following supporting information can be downloaded at https://www.mdpi.com/article/10.3390/ijms24086885/s1.
