# Supplementary material for: Growth-Restricted Fetuses and Offspring Reveal Adverse Sex-Specific Metabolic Responses in Preeclamptic Mice Expressing Human sFLT1
Source: Int J Mol Sci. 2023 Apr 7;24(8):6885. doi: 10.3390/ijms24086885 (PMC10139224; doi:10.3390/ijms24086885)
Supplement: Supplementary file 1 [file ijms-24-06885-s001.zip › ijms-2283089-supplementary.pdf]

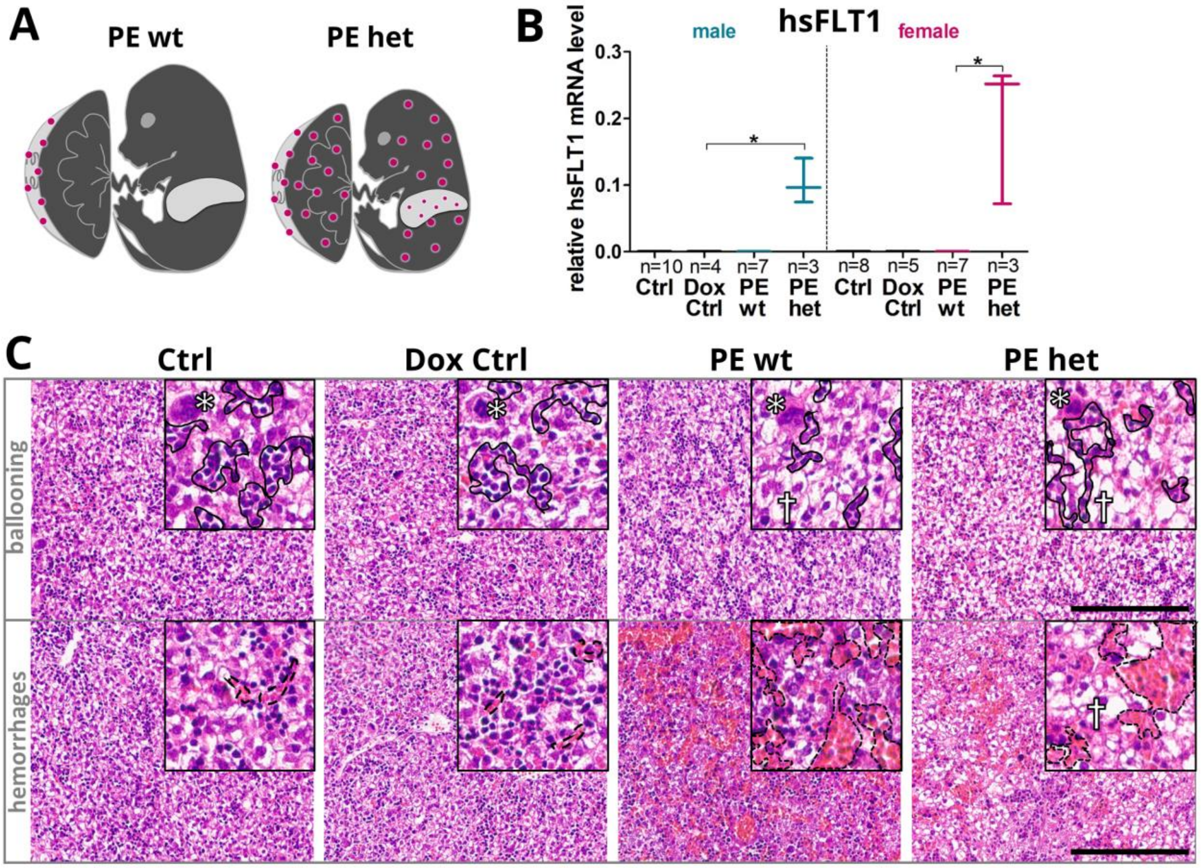

Figure S1: Fetal liver morphology upon systemic hsFLT1 overexpression at 18.5 dpc. A] Graphical presentation of the hsFLT1 expression in the PE wt and het fetuses. **B**] hsFLT1-mRNA expression analysis of fetal livers at the end of pregnancy (18.5 days post conception [dpc]) exhibit exclusive hsFLT1 overexpression in PE het fetuses in both sexes. Statistic was done with Kruskal Wallis and Dunn's multiple comparison post hoc test; *p<0.05. **C**] Fetal livers of all experimental groups showed presence of megakaryocytes and hematopoietic/erythropoietic islands distributed across the whole liver. Upon both types of hsFLT1 overexpression: exclusive maternal (PE wt) and combined maternal and feto-placental (PE het), fetal livers additionally showed signs of ballooning degeneration of hepatocytes, a histopathological sign of hepatocyte apoptosis observed as swelling of the hepatocytes combined with wispy cleared cytoplasm (upper panel). Additionally, PE wt and PE het fetal livers displayed regions of high erythrocyte presence (hemorrhages) in specifically affected parts of the liver. In fetal PE wt livers, ballooning and hemorrhages were mainly not seen in the same areas. Ballooning occurred in some fetal PE wt livers with high maternal hsFLT1 levels whilst hemorrhages were frequently seen, even upon lower maternal hsFLT1 levels. In fetal PE het livers, ballooning and hemorrhages often occurred commonly in the same liver. In both controls (Ctrl and Dox Ctrl) livers did not exhibit signs of increased bleeding or hepatocyte apoptosis. Asterisks (*): megakaryocytes; cross (†): ballooning, continuous lined areas: regions of hematopoiesis/ erythropoiesis, sectioned lined areas: regions with high erythrocyte presence/bleedings. Scale bar: 200 µm; Inserts in **C**]: 40x magification.

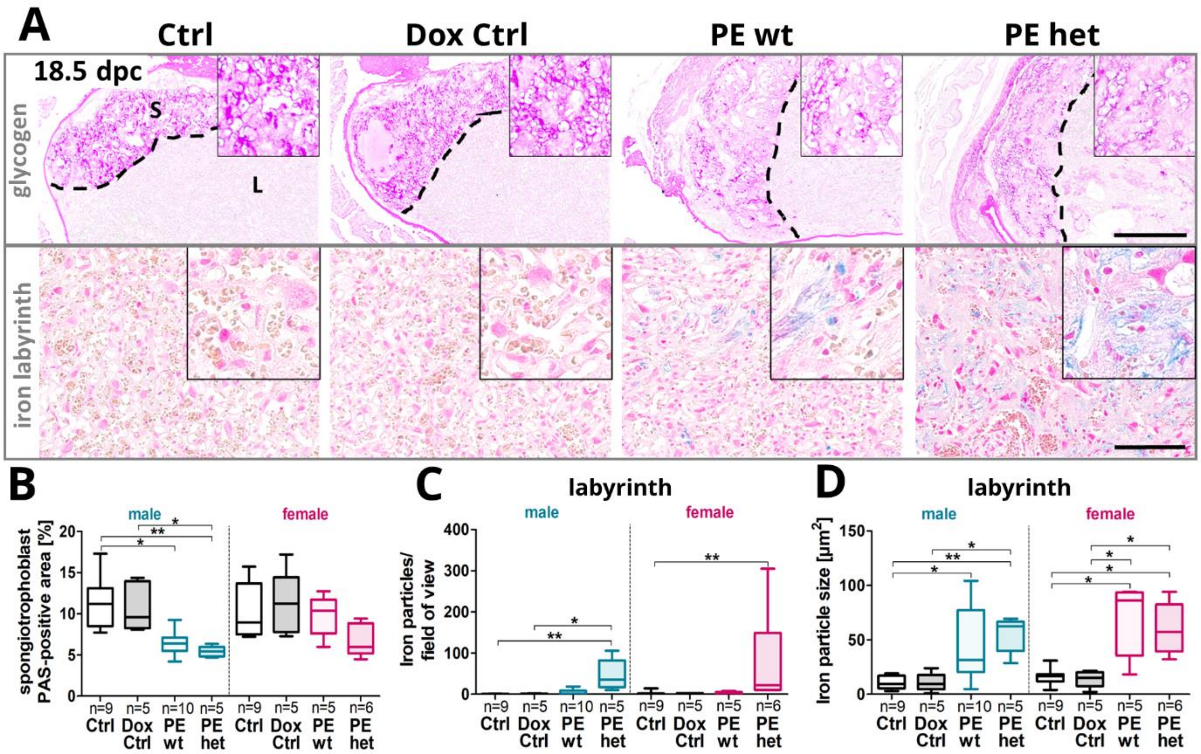

Figure S2: Reduced placental glycogen storage and increased iron deposit presence upon systemic hsFLT1 overexpression at 18.5 dpc. Consequences of maternal human soluble fms-like tyrosine kinase-1 (hsFLT1) upregulation on placenta morphology on 18.5 day post coitum (dpc). **A**] Periodic Acid Schiff's (PAS)-reaction was used to identify glycogen stores in the spongiotrophoblast layer of the placenta and iron deposits in the fetal labyrinth were detected by Perls Prussian Blue (Perls)-reaction. Scale bars: 300 µm for PAS and 100 µm for Perls). Inserts: 40x magification; L: Labyrinth; S: Spongiotrophoblasts. **B**] PAS-positive area in the spongiotrophoblast layer of the placenta was reduced upon hsFLT1 overexpression in PE wt and PE het males compared to controls (Ctrl and Dox Ctrl). **C-D**] Amount of iron particles over the field of view as well as the iron particle size was increased upon hsFLT1 overexpression in PE wt and especially PE het placentas. In both cases males were stronger affected than females. Data are presented as median, interquartile range and min/max. Sample size n is listed under each graph respectively for the tested placentas per group. Statistic was done with Kruskal Wallis and Dunn's multiple comparison post hoc test; $^{*}p<0.05$, $^{**}p<0.01$ and $^{***}p<0.001$.

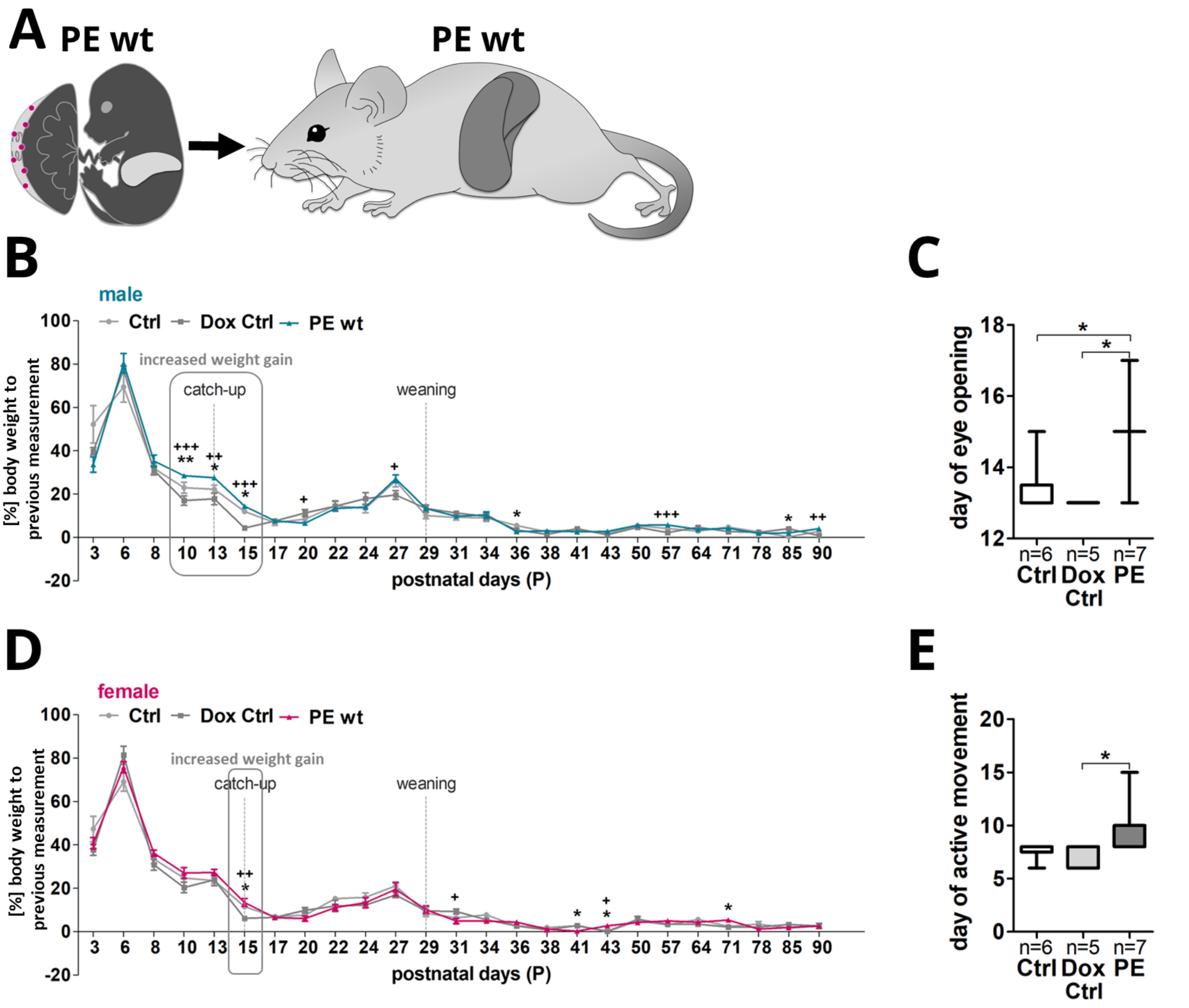

Figure S3: Postnatal weight gain from day of litter to postnatal day 90 (P90) measured as a percentage to the previous measurement. **A**] Consequences of previous hsFLT1 upregulation during pregnancy on postnatal weight gain and developmental progress. **B, D**] In addition to the absolute body weight, the weight gain as percent to the day before was calculated to reveal the time point of increased weight gain. In both sexes and all experimental groups, the highest percentual weight gain was observed from P3 to P6. Increased weight gain of hsFLT1-influenced offspring was seen in both sexes starting after P8 until P15, with male PE wt offspring [**B**] more pronounced than female [**D**]. **B]** After weaning, the difference in percentual weight gain was only increased in males at a few time-points. Although the weight gain was not often significantly different, it led to an increased absolute body weight at P90. **C, E**] Two milestones in postnatal development, **C**] the day of eye opening and **E**] the day of an active and independent walking/ movement, were analyzed at each day of weight measurement for the whole litter. Both, the day of eye opening [**C**], as well as the day of active movement [**E**] were delayed upon hsFLT1 overexpression during pregnancy. Statistic was done with Kruskal Wallis and Dunn's multiple comparison post hoc test; *p<0.05, **p<0.01 and ***p<0.001 and +p<0.05, ++p<0.01 and +++p<0.001 in B and D for comparison of Dox Ctrl to PE.

Table S1: Oligonucleotides used for quantitative real time polymerase chain reaction, genotyping and sex determination.

Gene	NCBI number	Primer sequence (5′ → 3′)	Base pairs
**Housekeeping genes**			
*β-Actin**	NM_007393.5	for: CCTCTATGCCAACACAGTGC	206
		rev: CCTGCTTGCTGATCCACATC	
Gapdh	XM_011241214.1	for: ACAACTCACTCAAGATTGTCAGCA	121
		rev: ATGGCATGGACTGTGGTCAT	
*Actinb-1**	NM_007393	for: AGCCATGTACGTAGCCATCCA	78
		rev: TCTCCGGAGTCCATCACAATG	
36b4	NM_007475	for: GCTTCATTG TGGGAGCAGACA	101
		rev: CATGGTGTTCTTGCCCATCAG	
**Angiogenesis**			
hsFLT1	XM_017020485.1	for: AATCATTCCGAAGCAAGGTG	221
		rev: TTTCTTCCCACAGTCCCAAC	
Flk-1	NM_001363216.1	for: GGCGGTGGTGACAGTATCTT	162
		rev: GTCACTGACAGAGGCGATGA	
Vegfa	NM_001025257.3	for: CAGGCTGCTGTAACGATGAA	140
		rev: GCATTCACATCTGCTGTGCT	
**Glucose and glycogen metabolism**			
Gyg	NM_001355261.1	for: TATACTCCTACCTCCCGGCA	223
		rev: TTGACAAGGCCATGGTGTTG	
Pygl	NM_133198.2	for: TACATTCAGGCTGTGCTGGA	203
		rev: AAGGCATCAAACACGGTTCC	
Gsk3b	NM_019827.7	for: GTCTGCTGGAGTACACACCT	245
		rev: AGTATCTGAGGCTGCTGTGG	
Lpk	NM_013631	for: CGTTTGTGCCACACAGATGCT<br>rev: CATTGGCCACATCGCTTGTCT	80
Glut2	NM_031197	for: AGAGGCATCGACTGAGCAGAA<br>rev: AGGATGGGCTGTCGGTAATTG	72
G6pc	NM_008061.2	for: CTGCAAGGGAGAACTCAGCAA<br>rev: GAGGACCAAGGAAGCCACAAT	67
Gk	NM_010292.3	for: CCTGGGCTTCACCTTCTCCTT<br>rev: GAGGCCTTGAAGCCCTTGGT	87

**Fatty acid metabolism**			
Scap	NM_001001144	for: CTGTGTACATTGATCAGACCATGG TA<br>rev: TGTTAGTACATC CCACAG GCAGAT	77
Srebp1c	NT_039515	for: GGAGCCATGGATTGCACATT<br>rev: CCTGTCTCACCCCCAGCATA	104
Srebp2	NM_033218.1	for: CTGCAGCCTCAAGTGCAAAG<br>rev: CAGTGTGCCATTGGCTGTCT	120
Fasn	NM_007988	for: GGCATCATTGGGCACTCCTT<br>rev: GCTGCAAGCACAGCCTCTCT	83
Ppara	NM_011144	for: TATTCGGCTGAAGCTGGTGTAC<br>rev: CTGGCATTTGTTCCGGTTCT	76
Lxra	NM_013839.2	for: TGCCTGATGTTTCTCCTGATTCT<br>rev: CCTCCCTGGTCTCCTGCAT	73
Lxrb	NM_009473	for: AAGGACTTCACCTACAGCAAGGA<br>rev: GAACTCGAAGATGGGATTGATGA	78
Insig2	NM_178082	for: GGGTATAAATCACGCCAGTGCTAA<br>rev: GGCCAAAACCACTTCTAGATCTGT	122
Elovl6	NM_130450.2	for: ACACGTAGCGACTCCGAAGAT<br>rev: AGCGCAGAAAACAGGAAAGACT	145
Elovl3	NM_001374665.1	for: GCCAAACTGAAGCATCCTAATCTT<br>rev: CCCAGAACCATCTGCAGAATC	71
**Genotyping**			
hsFLT1	NM_001159920.2	for: CAAGGACGTAACTGAAGAGG	465
		rev: TTTCTTCCCACAGTCCCAAC	
Col1a1		for: CCATCCCAACAATACATCACA	200
		rev: TGGTTTCTTTGGGCTAGAGG	
rtTA		for: AAAGTCGCTCTGAGTTGTTAT	
		rev-wt: GGAGCGGGAGAAATGGATATG	650
		rev-mut: GCGAAGAGTTTGTCCTCAACC	340
**Sex determination**			
IL-3	NM_010556.4	for: GGGACTCCAAGCTTCAATCA	544
		rev: TGGAGGAGGAAGAAAAGCAA	
Sry	NM_011564.1	for: TGGGACTGGTGACAATTGTC	402
		rev: GAGTACAGGTGTGCAGCTCT	
